# Preclinical studies show using enzalutamide is less effective in docetaxel-pretreated than in docetaxel-naïve prostate cancer cells

**DOI:** 10.18632/aging.103917

**Published:** 2020-09-10

**Authors:** Changyi Lin, Fu-Ju Chou, Jieyang Lu, Wanying Lin, Matthew Truong, Hao Tian, Yin Sun, Jie Luo, Rachel Yang, Yuanjie Niu, Rosa Nadal, Emmanuel S. Antonarakis, Carlos Cordon-Cardo, Deepak Sahasrabudhe, Chi-Ping Huang, Shuyuan Yeh, Gonghui Li, Chawnshang Chang

**Affiliations:** 1 George Whipple Lab for Cancer Research, Departments of Pathology, Urology, Radiation Oncology and The Wilmot Cancer Institute, University of Rochester, Rochester, NY 14642, USA; 2 Department of Urology, Sir Run Run Shaw Hospital, Zhejiang University School of Medicine, Hangzhou 310016, China; 3 Chawnshang Chang Sex Hormone Research Center, Tianjin Institute of Urology, Tianjin Medical University, Tianjin 300211, China; 4 Sidney Kimmel Comprehensive Cancer Center at Johns Hopkins, Baltimore, MD 21231, USA; 5 Department of Pathology, Mount Sinai School of Medicine, New York, NY 10029, USA; 6 Sex Hormone Research Center, Department of Urology, China Medical University and Hospital, Taichung 404, Taiwan

**Keywords:** docetaxol, enzalutamide, ARv7, prostate cancer, resistance

## Abstract

Anti-androgen therapy with Enzalutamide (Enz) has been used as a therapy for castration resistant prostate cancer (CRPC) patients after development of resistance to chemotherapy with Docetaxel (Doc). The potential impacts of Doc-chemotherapy on the subsequent Enz treatment, however, remain unclear. Here we found the overall survival rate of patients that received Enz was significantly less in patients that received prior Doc-chemotherapy than those who had not. *In vitro* studies from 3 established Doc resistant CRPC (DocRPC) cell lines are consistent with the clinical findings showing DocRPC patients had decreased Enz-sensitivity as well as accelerated development of Enz-resistance *via* enhanced androgen receptor (AR) splicing variant 7 (ARv7) expression. Mechanism dissection found that Doc treatment might increase the generation of ARv7 *via* altering the MALAT1-SF2 RNA splicing complex. Preclinical studies using *in vivo* mouse models and *in vitro* cell lines proved that targeting the MALAT1/SF2/ARv7 axis with small molecules, including siMALAT1, shSF2, and shARv7 or ARv7 degradation enhancers: Cisplatin or ASC-J9^®^, can restore/increase the Enz sensitivity to further suppress DocRPC cell growth. Therefore, combined therapy of Doc-chemotherapy with anti-ARv7 therapy, including Cisplatin or ASC-J9®, may be developed to increase the efficacy of Enz to further suppress DocRPC in patients.

## INTRODUCTION

Chemotherapy with Docetaxel (Doc) has been established as the standard-of-care to treat patients with castration resistant prostate cancer (CRPC) at the metastatic stage [1, 2]. Mechanism dissections suggested that the anti-neoplastic effect of Doc could be mainly through a result of anti-mitotic effects [3]. However, other studies indicated that Doc might interrupt microtubule-dependent trafficking of androgen receptor (AR) into the nuclei, and clinical data also linked cytoplasmic AR in circulating tumor cells with patients’ responses to Doc-chemotherapy.

The average period of Doc-chemotherapy efficacy is 18 months before development of Doc-chemotherapy resistance (Doc-resistance). The androgen-deprivation-therapy (ADT) with Enzalutamide (Enz) may extend survival of CRPC patients by several more months [4, 5] before development of Enz-resistance. Enz is a recently developed powerful anti-androgen that can prevent androgens from binding to AR, the key master influencing PCa progression [6–9]. The failure of Enz has been linked to the development of AR splicing variants, including ARv7 [10]. However, the potential linkage of pre-Doc-chemotherapy to the efficacy of subsequent Enz treatment remains unclear.

We found that Doc-chemotherapy may induce adverse effects *via* increasing the ARv7 expression, which may then accelerate the development of Enz-resistance, thus reducing the efficacy of subsequent Enz treatment. Therefore, the potential combination of Doc-chemotherapy plus anti-ARv7 therapy may help increase the efficacy of subsequent Enz treatment to suppress Enz-resistant (EnzR) cell growth.

## RESULTS

### Human clinical data showing Enz is less effective in Doc-pretreated CRPC patients compared to Doc-naive CRPC patients

After PCa patients developed CRPC, the current standard therapy involves either Doc-chemotherapy or ADT with either Enz or Abiraterone [11]. The potential impact of pre-Doc-chemotherapy (after developing Doc-resistance) to subsequent Enz treatment, however, remains unclear.

We examined whether Doc-chemotherapy reduces the efficacy of subsequent Enz treatment, and retrospectively examined clinical outcomes in 114 consenting patients with metastatic CRPC that received Enz at Johns Hopkins Hospital between March 2012 and August 2014. Of those, 52 men received Enz prior to Doc-chemotherapy (Doc-naïve) and 62 men received Enz following Doc-chemotherapy (Doc-pretreated). We investigated prostate specific antigen (PSA) response rates, PSA progression-free survival (PSA-PFS), progression-free survival (PFS), and overall survival (OS); comparing outcomes in Doc-naïve patients *vs* Doc-pretreated patients. PSA response was defined as 50% PSA decline from baseline during Enz treatment, PSA-PFS as the length of time from Enz initiation until time of 25% increase from baseline/nadir value, PFS as the length of time from Enz initiation until clinical or radiographic disease progression, and overall survival (OS) as the length of time from Enz initiation until death from any cause.

As shown in the Table 1, all clinical outcomes to Enz were significantly poorer in men who had previously received Doc-chemotherapy compared to untreated patients showing cross-resistance between Doc-chemotherapy and subsequent Enz treatment.

**Table 1:** Baseline characteristics of enzalutamide-treated patients, by prior docetaxel treatment.

	**Docetaxel-naïve (n=52)**	Docetaxel-pretreated^*^ (n=62)	***P* value**
**Race, N (%)**			
**Non-AA**	46 (89)	53 (85)	0.54
**AA**	6 (11)	9 (15)	
**Gleason sum, N (%)**			
**≤ 7**	25 (48)	22 (35)	
**≥ 8**	27 (52)	40 (65)	0.17
**T stage, N (%)**			
**T1/T2**	20 (38)	21 (34)	
**T3/T4**	32 (62)	41 (66)	0.62
**M stage at diagnosis, N (%)**			
**M0**	37 (71)	46 (74)	
**M1**	15 (29)	16 (26)	0.82
Prior anti-androgen therapy^**^,			
**median N (%)**	2 (1 – 4)	2 (1 – 5)	0.76
**ECOG performance status, N (%) ^#^**			
**0**	29 (55)	30 (48)	
**≥1**	23 (45)	32 (52)	0.65
**Bone pain, N (%)**			
**No**	30 (58)	28 (45)	
**Yes**	22 (42)	34 (55)	0.28
**Visceral metastases, N (%)**			
**No**	49 (94)	51 (82)	
**Yes**	3 (6)	11 (18)	0.04
**Hemoglobin level, g/dL, mean (SD)**	12.1 (±2.0)	11.3 (±1.8)	0.07
**Albumin level, g/dL, mean (SD)**	4.3 (±1.0)	4.0 (±0.4)	0.16
**Alkaline phosphatase level, IU/L, mean (SD)**	158 (±211)	167 (±170)	0.19

AA: African-American, ECOG: Eastern Cooperative Group, SD: standard deviation, PSA: prostate-specific antigen.

^*^Patients treated with docetaxel before enzalutamide.

^**^Anti-androgen therapy refers to a first-generation androgen-receptor antagonist (*i.e.* flutamide, bicalutamide, nilutamide) or ketoconazole.

^#^There were no patients with ECOG performance status 4. Only 2 patients in the Docetaxel-pretreated group and 1 patient in the Docetaxel-naïve group had a performance status of 3.

### Development of Doc-resistance in PCa cells altered the sensitivity of subsequent Enz treatment

To confirm human clinical data in *in vitro* CRPC cells, we first established DocR CRPC (DocRPC) cell lines from CWR22Rv1 (named DocR1_CWR22Rv1) and C4-2 cells (named DocR3_C4-2), (see detailed procedure in Materials and Methods). We obtained another DocR_CWR22Rv1 cell line (named as DocR2-CWR22Rv1) from Dr. Carlos Cordon-Cardo [12]. These 2 DocR CWR22Rv-1 cell lines were generated by different treatment schedules.

**Figure 1:**
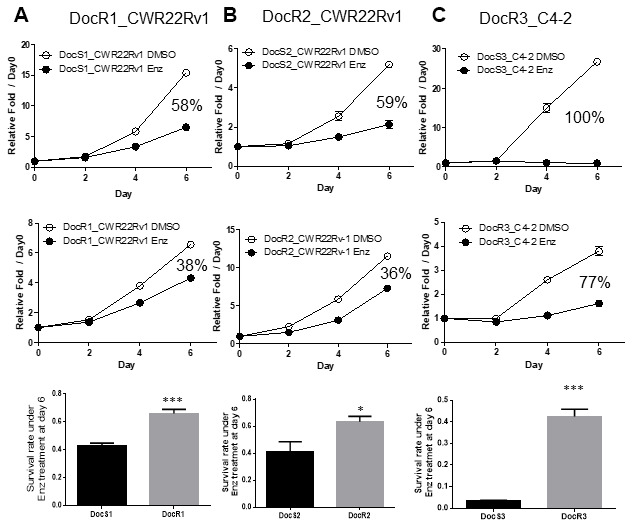
**Acquired Enz-resistance after Doc-resistance in DocRPC cells.** (**A**) The growth curve of DocR1_CWR22Rv1 cells after 6 days 20 μM Enz treatment. DocR1_CWR22Rv1 cells (middle) are more resistant to Enz treatment than DocS1_CWR22Rv1 cells (upper), with 38% vs 58% growth reduction. Survival rate (lower) under Enz treatment at Day 6 (DocR1 *vs* DocS1 cells). (**B**) The growth curve of DocR2_CWR22Rv1 cells after 6 days 20 μM Enz treatment. DocR2_CWR22Rv1 cells (middle) are more resistant to Enz treatment than DocS1_CWR22Rv1 cells (upper), with 36% *vs* 59% reduction. Survival rate (lower) under Enz treatment at Day 6 (DocR2 *vs* DocS2 cells). (**C**) The growth curve of DocR3_C4-2 cells after 6 days 15 μM Enz treatment. DocR3-C4-2 cells (middle) are more resistant to Enz treatment than DocS3_C4-2 cells (upper), with 77% vs 100% reduction. Survival rate (lower panel) under Enz treatment at Day 6 (DocR3 vs DocS3 cells). Results are mean ± SD compared to controls *p<0.05; ***p<0.001.

Using MTT growth assay to compare the sensitivities to subsequent Enz treatment in these DocRPC cells, we found DocR1-CWR22Rv1 cells are more resistant to Enz as compared to their parental Doc-sensitive (DocS) cells (day 6 38% *vs* 58%, Figure 1A and Supplementary Figure 1A). Similar results were obtained when we replaced DocR1_CWR22Rv1 cells with two other DocR cells lines (DocR2_CWR22Rv1; day 6 36% *vs* 59%, Figure 1B; and DocR3_C4-2; day 6 77% *vs* 100%, Figure 1C and Supplementary Figure 1B).

Together, results from Figure 1A–1C and Supplementary Figure 2A, 2B suggest that Doc-resistance developed in multiple CRPC cells may decrease subsequent Enz sensitivity to suppress DocRPC cell growth.

### Acquired Doc-resistance in CRPC cells promotes DocRPC cells to develop Enz-resistance

**Figure 2:**
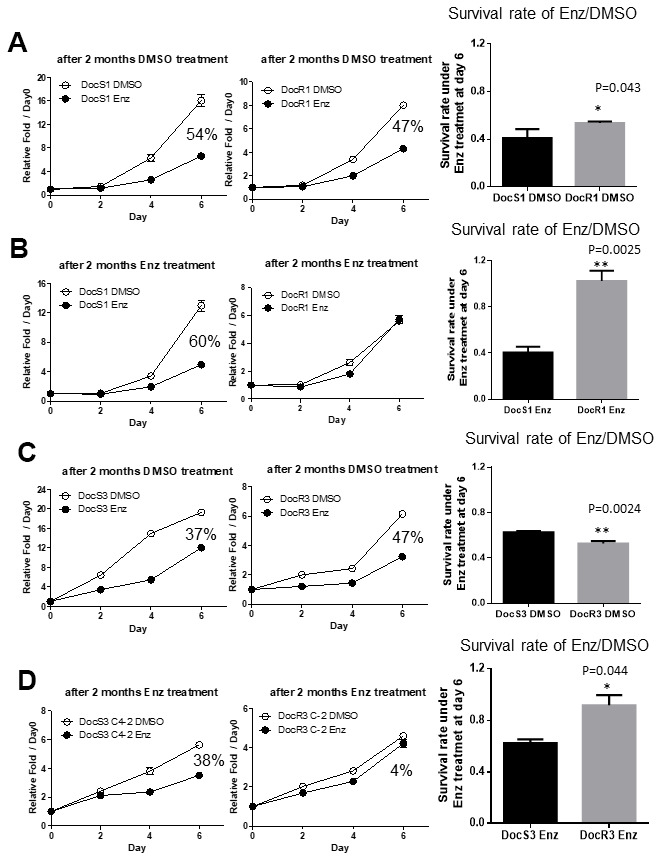
**Acquired Doc-resistance increases DocRPC cells development of Enz-resistance after two months of Enz treatment.** (**A**) DocS1_CWR22Rv1 (left) and DocR1_CWR22Rv1 (middle) cells were cultured with DMSO for 2 months and then assayed for Enz sensitivity after treated with/without 20 μM Enz for 2, 4 and 6 days. DocR1_CWR22Rv1 cells have slightly less Enz sensitivity (47% *vs* 54%) compared to DocS1_CWR22Rv1 cells. (**B**) DocS1_CWR22Rv1 (left) and DocR1_CWR22Rv1 (middle) cells were continually cultured with 20 μM Enz for 2 months, and the cell growth was examined after treating with/without Enz for 0, 2, 4 and 6 days. DocR1_CWR22Rv1 cells have much less Enz sensitivity (from 60% to 1%) compared to DocS1_CWR22Rv1 cells. (**C**) DocS3_C4-2 (left) and DocR3_C4-2 (middle) cells were cultured with DMSO for 2 months and then were assayed for Enz sensitivity after treating with/without DMSO/Enz for 2, 4 and 6 days. DocR3-C4-2 cells have similar Enz sensitivity (37% *vs* 47%) compared to DocS3_C4-2. (**D**) DocS3_C4-2 (left) and DocR3_C4-2 (middle) were continually cultured with 20 μM Enz for 2 months, and the cell growth was examined after treating with/without Enz for 0, 2, 4 and 6 days. DocR3_C4-2 have less Enz sensitivity (from 38% to 4%) comparing with DocS3_C4-2. For A-D the survival rates of 10 μM Enz/DMSO at day 6 are shown in right panels. Results are mean ± SD compared to controls *p<0.05; **p<0.01.

In addition to decreasing sensitivity to subsequent Enz treatment, we were interested to see if Doc-resistance may also accelerate the development of Enz-resistance in DocRPC cells. We compared the Enz sensitivity between DocR1_CWR22Rv1 *vs* their parent DocS1_CWR22Rv1 cells by treating with DMSO or 20 μM Enz for 2, 4 and 6 days, and MTT analysis results revealed both cell lines were sensitive to Enz treatment with DocS1_CWR22Rv1 having slightly higher sensitivity than DocR1_CWR22Rv1 (54% vs 47% in Figure 2A). We then continuously treated DocS1_and DocR1_CWR22Rv1 with/without 20 μM Enz for two months, and again tested their Enz-sensitivity. The results revealed that DocS1_CWR22Rv1 cells still retained similar Enz sensitivity (at 60% in Figure 2B). In contrast, DocR1_CWR22Rv1 cells lost Enz sensitivity (1% in Figure 2B).

Similar results were obtained when we replaced DocS1_CWR22Rv1 and DocR1_CWR22Rv1 with DocS3_C4-2 and DocR3_C4-2 cells treated with/without 20 μM Enz for 2 months showing DocS3_C4-2 retained similar Enz sensitivity (37% to 38% in Figure 2C left and Figure 2D left), yet DocR3_C4-2 had significantly reduced Enz sensitivity (47% to 4% in Figure 2C middle and Figure 2D middle) after the 2, 4, and 6 days of testing for MTT assays. Together, results from Figure 2A–2D demonstrated the Doc-resistance developed in the DocRPC cells could also result in accelerated development of Enz-resistance, which might decrease the subsequent Enz treatment efficacy.

### Mechanism dissection how Doc-resistance promotes DocRPC cells to develop Enz-resistance

**Figure 3:**
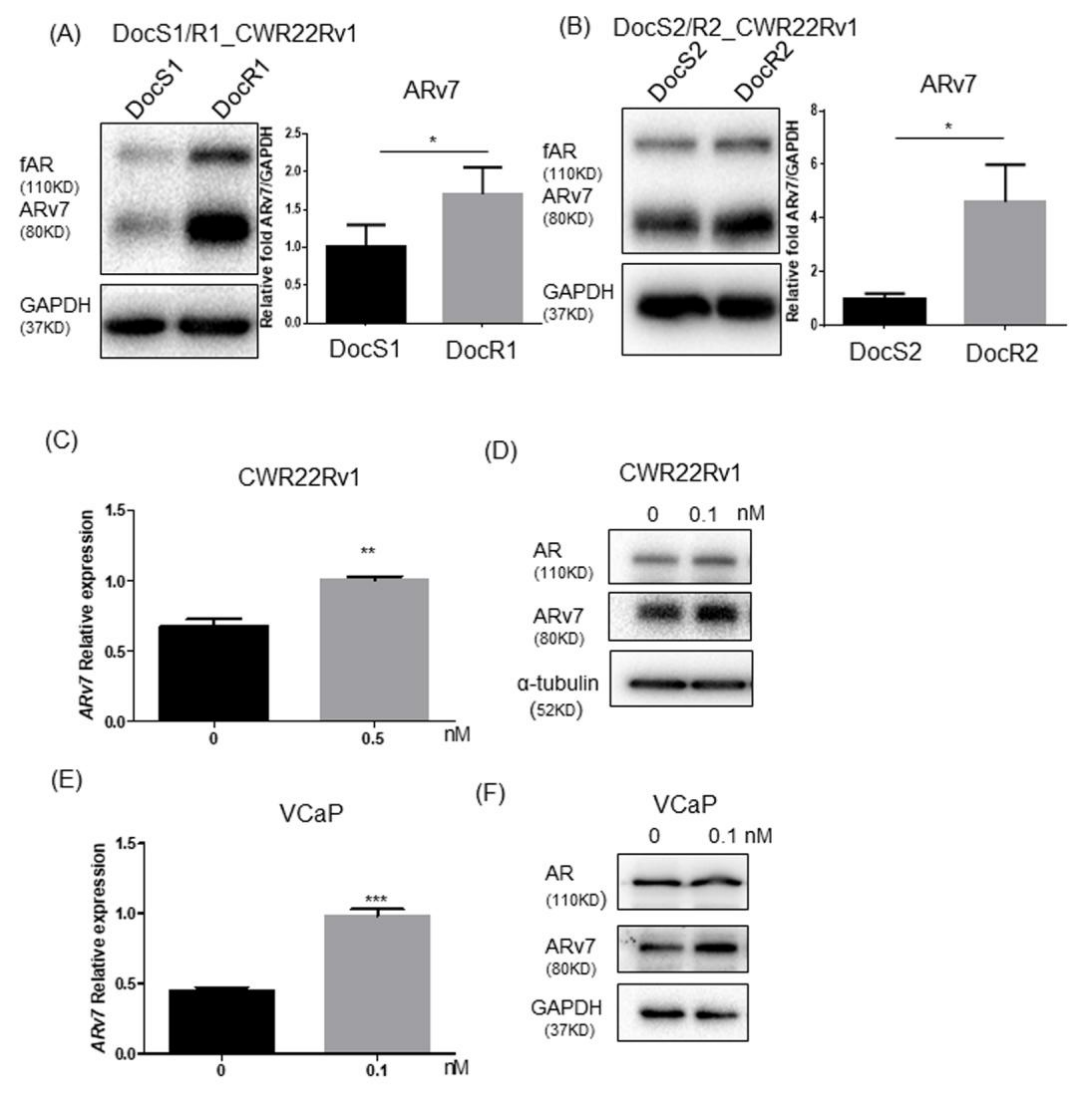
**Doc treatment increased ARv7 in CRPC and DocRPC cells.** (**A**, **B**) ARv7 expression (left panel: immunoblot for protein level; right panel: real-time PCR for mRNA level) is higher in DocR1_ and DocR2_CWR22Rv1 compared to DocS1_ and DocS2_CWR22Rv1 cells. (**C**–**F**) Increasing ARv7 expressions at protein levels and mRNA levels upon transient Doc treatment in Doc naïve parental CWR22Rv1 (**C**, **D**) and VCaP (**E**, **F**) cells. After treating parental CWR22Rv1 or VCaP cells with DMSO or Doc, then ARv7 mRNA (**C**, **E**) and ARv7 protein (**D**, **F**) were assayed. Results are mean ± SD compared to controls, *p<0.05; **p<0.01; ***p<0.001.

Recent clinical studies from CRPC patients receiving Enz found that patients with higher ARv7 expression in their CRPC tumors may poorly respond to Enz treatment, which might also enhance the ARv7 expression in their tumors, suggesting ARv7 may play key roles for development of Enz resistance [13, 14]. We first examined the Doc effects on ARv7 expression in CRPC cells and found the expression of both full-length AR (fAR) and ARv7 were increased in DocR CRPC cells compared to their parental CRPC cells that are sensitive to DocS (DocR1_CWR22Rv1, ARv7 level in Figure 3A with Doc sensitivity in Supplementary Figure 2A, DocR2_CWR22Rv1, ARv7 level in Figure 3B with Doc sensitivity in Supplementary Figure 2B, DocR3_C4-2 with Doc sensitivity in Supplementary Figure 2C, and ARv7 level in Supplementary Figure 2E, and DocR4_VCaP with Doc sensitivity in Supplementary Figure 2D and ARv7 level in Supplementary Figure 2F), suggesting increased ARv7 expression may be linked to Doc effects on the development of Enz-resistance during subsequent Enz treatment.

We then treated CWR22Rv1 cells with Doc for 24 hrs and found Doc could enhance ARv7 expression at both mRNA (Figure 3C) and protein levels (Figure 3D). Similar results were also obtained when we replaced CWR22Rv1 cells with VCaP cells (Figure 3E, 3F), suggesting adding Doc alone is sufficient to enhance ARv7 expression.

Together, results from Figure 3A–3F and Supplementary Figure 2 suggest that Doc treatment can enhance the expression of ARv7, a key factor contributing to development of Enz resistance in CRPC cells.

### Mechanism dissection how Doc increased ARv7 expression in CRPC cells

**Figure 4:**
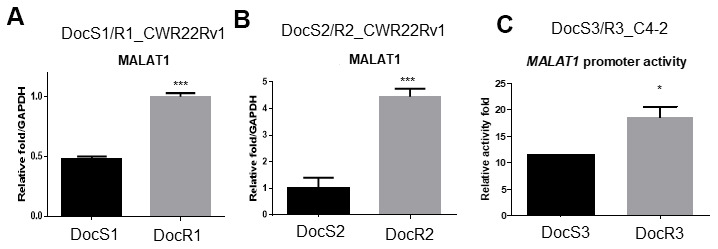
**Doc increased the expression of MALAT1 in DocR- cells.** (**A**–**C**) The RNA expression of MALAT1 (**A**) in DocS1_CWR22Rv1 and DocR1_CWR22Rv1 cells, (**B**) in DocS2_CWR22Rv1 and DocR2_CWR22Rv1 cells, (**C**) *MALAT1* promoter activity in DocR3_C4-2 cells compared to DocS3_C4-2 cells. Results are mean ± SD compared to controls, *p<0.05; ***p<0.001.

To dissect the mechanism(s) of how Doc increases ARv7 expression in CRPC cells, we first examined the factors involved in RNA splicing as early studies indicated ARv7 might be spliced from fAR [7, 10] involving the RNA splicing process [15]. The results revealed the expression of Metastasis Associated Lung Adenocarcinoma Transcript 1 (MALAT1) (Gene ID:378938), a lncRNA [16–18] that is a the key molecule in RNA splicing [19, 20], as well as ARv7, was higher in DocR1_CWR22Rv1 as compared to DocS1_CWR22Rv1 parental cells (Figure 4A). We obtained similar data when we replaced DocR1_CWR22Rv1 with DocR2_CWR22Rv1 cells (Figure 4B).

To further dissect the mechanism at molecular levels, we linked the *MALAT1* 5′ promoter region to a luciferase reporter and assayed the Doc effects. The results showed Doc treatment could increase MALAT1 expression at the transcriptional level (Figure 4C) in DocR3_C4-2 cells.

Together, results from Figure 4A–4C suggest Doc treatment may enhance ARv7 *via* increasing expressions of RNA splicing complex member, MALAT1.

### Targeting MALAT1/SF2/ARv7 axis with siRNAs/shRNAs or Cisplatin/ASC-J9^®^ increased the efficacy of Enz to further suppress the DocR-CWR22Rv1 cells growth

Results from Figures 1–4 demonstrate Doc-chemotherapy can increase expression of the MALAT1/SF2 RNA splicing complex to increase ARv7 expression to accelerate development of Enz-resistance in DocRPC cells. These findings may have significant clinical implications if we can target the MALAT1/SF2/ARv7 axis to delay or even eliminate the development of Enz-resistance in DocRPC cells.

**Figure 5:**
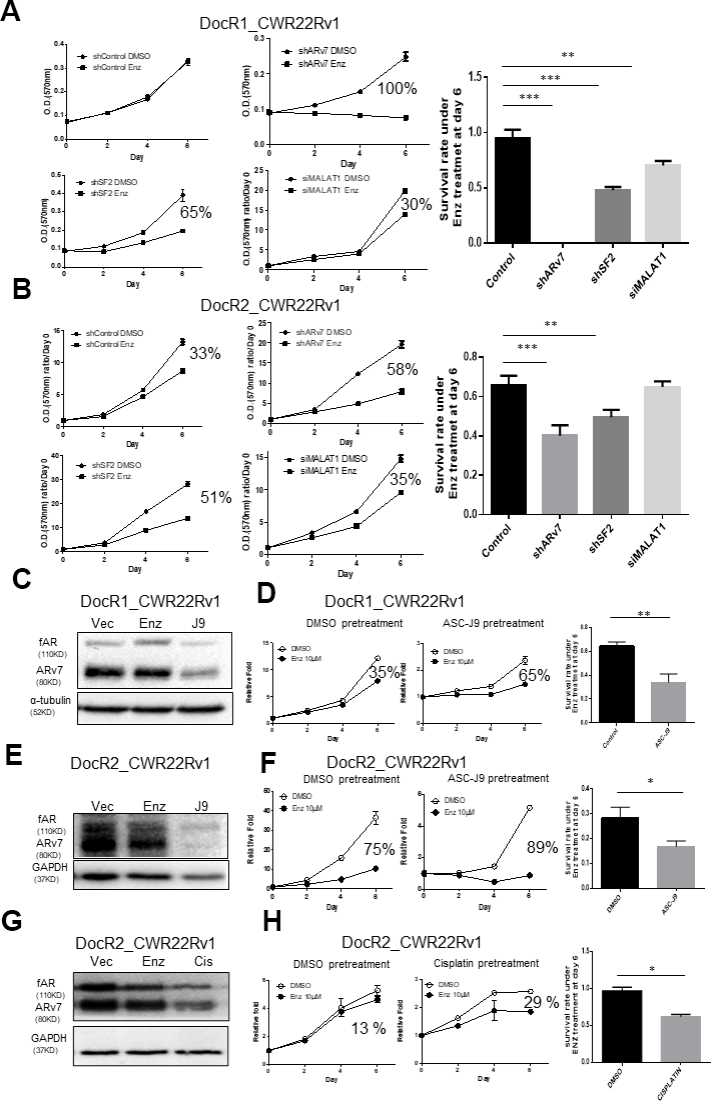
**Targeting MALAT1/SF2/ARv7 axis increased the efficacy of Enz to further suppress the DocR_22Rv1 cells growth.** (**A**, **B**) The growth curve of DocR1_CWR22Rv1 (**A**) and DocR2_CWR22Rv1 (**B**) cells in response to Enz treatment after targeting MALAT1/SF2/ARv7 axis with either ARv7-shRNA (upper middle), SF2-shRNA (lower left), or MALAT1-siRNA (lower middle). Right panels are quantification of survival rate under Enz treatment at Day 6. (**C**–**H**) The protein level of (**C**, **E**) fAR and ARv7, 6 days growth curves (**D** and left) and statistics (**D** and **F** right) of Day 6 (right) of (**C**, **D**) DocR1_CWR22Rv1 after DMSO, 10 μM Enz, or 5 μM ASC-J9^**®**^ treatment for 24 hrs and (**E**, **F**) DocR2_CWR22Rv1 after DMSO, 10 μM Enz, or 5 μM ASC-J9^**®**^ treatment for 24 hrs. (**G**, **H**) DocR2_CWR22Rv1 protein levels (**G**) growth curves after DMSO, 10 μM Enz or 3 μg/ml Cisplatin (CIS) treatment (**H**) for 24 hrs. Results are mean ± SD compared to controls *p<0.05; **p<0.01; ***p<0.001.

We targeted the MALAT1/SF2/ARv7 axis to suppress ARv7 expression in DocR1_CWR22Rv1 cells with/without either MALAT1-siRNA, SF2-shRNA, or ARv7-shRNA, SF2-shRNA, or MALAT1-siRNA (efficiency see Supplementary Figure 3A–3C) and results revealed the suppression of ARv7 restored/increased cells sensitivity in response to Enz treatment (ARv7-shRNA: near 100%; SF2-shRNA: 65%, and MALAT1-siRNA: 30%, in Figure 5A) compared to controls. Similar results were obtained when we replaced DocR1_CWR22Rv1 cells with DocR2_CWR22Rv1 (shARv7: from 33% to 58%; SF2-shRNA: from 33% to 51%, and MALAT1-siRNA: from 33% to 35%, in Figure 5B).

We also applied the recently developed AR degradation enhancer, ASC-J9^®^, that could degrade both AR and ARv7 with little adverse effects [21–27] to examine its effect on Doc-induced Enz resistant cells. The results revealed pretreating with 10 μM ASC-J9^®^ suppressed ARv7 in DocR1_CWR22Rv1 cells (Figure 5C), which restored/increased DocR1_CWR22Rv1 cell sensitivity to Enz (from 35% to 65%, Figure 5D). Similar results were obtained when we replaced DocR1_CWR22Rv1 cells with DocR2_CWR22Rv1 (from 75% to 89%, Figure 5E, 5F)

Additionally, using Cisplatin that could also degrade AR and ARv7 (Figure 5G and Supplementary Figure 4A), we obtained similar results showing Cisplatin treatment could restore/increase Enz sensitivity to further suppress DocRPC cell growth (Figure 5H; from less than 13% to 29%, Supplementary Figure 4B; from 14% to 22%).

Together, results from *in vitro* cell lines studies (Figure 5A–5H) demonstrated targeting the MALAT1/SF2/ARv7 axis via MALAT1-siRNA, SF2-shRNA, ARv7-shRNA, ASC-J9^®^, or cisplatin could restore/increase Enz sensitivity to further suppress DocRPC cell growth.

### Preclinical study using *in vivo* mouse model to prove targeting MALAT1/SF2/ARv7 axis with ARv7-shRNA or ASC-J9^®^ can further suppress DocRPC cell growth

**Figure 6:**
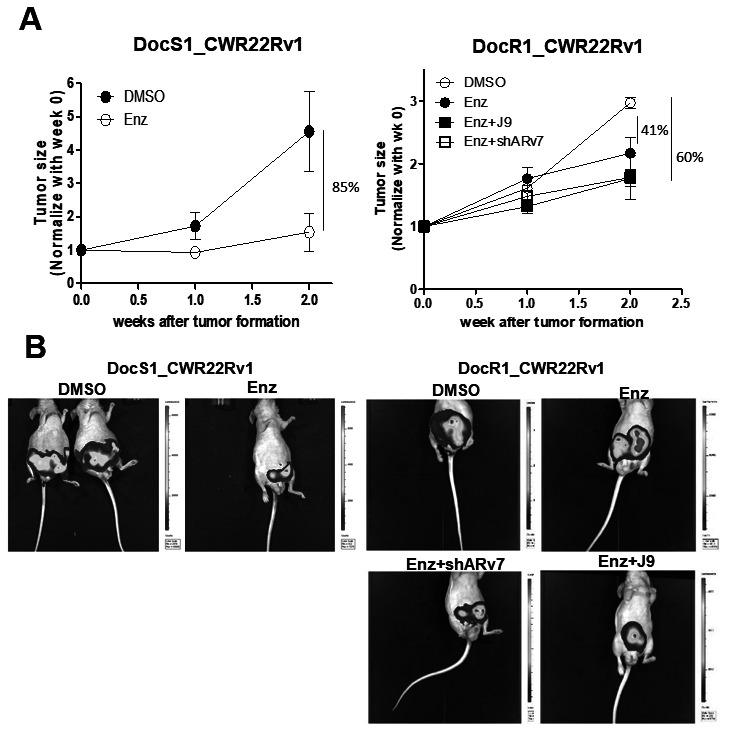
**Targeting ARv7 with ARv7-shRNA or ASC-J9^®^ further suppressed the DocR1-CWR22Rv1 cell growth in the *in vivo* mouse model.** DocS1_CWR22Rv1, DocR1_CWR22Rv1 (left panel), DocR1_CWR22Rv1+shControl and DocR1_CWR22Rv1+shARv7 (right panel) cells (1x10^6^ cells) were labeled with luciferase and injected orthotopically into nude mice. Two weeks after implantation, mice were treated with DMSO, Enz, or Enz+ASC-J9^**®**^ for 2 weeks. Tumors were formed and visualized by IVIS image. (**A**) Growth curve of tumor size after tumor formation in groups (n=3 in each group). (**B**) Representative IVIS images of each group’s end point.

To prove *in vitro* cell lines results in the *in vivo* mouse model, we orthotopically xenografted DocS1_CWR22Rv1 and DocR1_CWR22Rv1 cells (with stable transfected luciferase for IVIS imaging) into the anterior prostates of nude mice for the following 6 treatment groups: (1) DocS1_CWR22Rv1 + DMSO; (2) DocS1_CWR22Rv1 + Enz; (3) DocR1_CWR22Rv1 + DMSO; (4) DocR1_CWR22v1 + Enz; (5) DocR1_CWR22Rv1 transduced shARv7 + Enz; and (6) DocR1_CWR22RV1 + Enz and ASC-J9^®^ (Figure 6).

As expected, the *in vivo* mice results matched well with the *in vitro* cell lines results showing Enz treatment has less effect on Doc-resistance tumor growth (41%) compared to the Doc-sensitive tumor growth (85%) (Figure 6A, 6B left). Additionally, targeting ARv7 with ARv7-shRNA or ASC-J9^®^ has the tendency to restore the Enz sensitivity in the DocR groups (from 41% to 61% for ARv7-shRNA and from 41% to 60% for ASC-J9^®^, Figure 6A, 6B right).

Together, results from preclinical studies using *in vivo* mouse model (Figure 6) demonstrated development of Doc-resistance may alter the efficacy of subsequent Enz treatment and targeting this Doc-induced MALAT1/SF2/ARv7 axis with shARv7 or ASC-J9^®^ might help DocRPC tumors to have restored/increased sensitivity to subsequent Enz treatment to further suppress cell growth.

## DISCUSSION

A previous study indicated that applying Enz in CRPC patients after chemotherapy could increase patients’ survival [4, 11]. This is important to some countries, including China, that are beginning to have clinical trials of using Enz treatment to treat CRPC patients who failed to respond to other therapies. Doc is an effective chemotherapy for CRPC patients but may also have some adverse effects that may impact the subsequent Enz treatments. Detailed mechanisms contributing to cross-resistance are not well understood. We demonstrated that DocRPC cells could develop Enz-resistance more easily compared to their parental cells *in vitro* and *in vivo*. We also uncovered that the AR variant, ARv7, might play a critical role in Doc and Enz cross-resistance. Targeting Doc-increased ARv7 *via* shARv7, or siMALAT1 and using AR degradation enhancers, Cisplatin or ASC-J9^®^, could restore/increase the Enz sensitivity in cells and in the mouse model studies. These results suggested Doc could increase ARv7 expression and make CRPC cells become cross-resistant to subsequent Enz treatment through altering the MALAT1/SF2/ARv7 signals. It will be of future importance to see if targeting these altered MALAT1/SF2/ARv7 signals can prolong the Enz efficacy, including better PSA response rates and clinical radiographic PFS.

Early studies indicated that some molecules might be involved in the development of DocR [12, 28–30] and EnzR [13, 14, 31] cell lines. However, there have been no reports indicating which molecule could overcome subsequent Enz resistance in patients that previously developed Doc-resistance. Here we demonstrated that ARv7, which can be induced by Doc treatment, has a key role and can further alter the efficacy of subsequent Enz treatment in CRPC cells. Interestingly, in the recently reported ARMOR3-SV Clinical Trial comparing Enz *vs* galeterone, the prevalence of ARv7 detection in CRPC patients was 5.8% in Doc-naïve patients and 22.4% in Doc-pretreated patients [14], supporting the hypothesis that prior Doc treatment may increase ARv7-positive disease.

ARv7 has a few unique sequences derived from cryptic exons or from out-of-frame translation of full-length AR. This AR splicing variant is constitutively active and reportedly activates/inhibits a transcriptional program that is similar, but not identical, to that of AR in CRPC [26, 32–34] cells. Tripathi et al. found that the lncRNA-MALAT1 could interact with SF2 and we demonstrated that it is critical for ARv7 expression [20]. A previous study indicated targeting MALAT1 could inhibit PCa cell growth [35]. This evidence suggested that the MALAT1/SF2/ARv7 axis might have key roles for increased ARv7 splicing and development of Enz resistance. Our findings confirmed this concept, and for the first time demonstrated the MALAT1/SF2-ARv7 axis is the common pathway in charge of both Doc and subsequent Enz cross-resistance development.

Alternative splicing of some key genes may occur as a result of differential spliceosome assembly in response to cellular stress, such as oxidative stress, hypoxia [36], or genotoxic stress [37, 38]. Our data showed transient Doc treatment can increase SF2 phosphorylation (Supplementary Figure 5) and change SF2 activity and function [39]. We speculate Doc-chemotherapy might increase intracellular stress by inhibiting cell division and trigger spliceosome assembly containing MALAT1 and phosphorylated SF2, which could recognize the cryptic splicing junctions to produce ARv7 mRNA from the AR gene precursor RNA transcript.

The involvement of MALAT1 in ARv7 expression highlights the role of nuclear lncRNAs in gene expression regulation. Early studies indicated MALAT1 actively regulates gene expression, including a set of metastasis-associated genes in lung cancer [40]. MALAT1-siRNA not only can suppress the splicing of ARv7, but can also suppress the expression of those MALAT1-regulated metastasis genes.

Compared to the potential delivery difficulty and toxicity of using siRNAs/shRNAs to target the MALAT1/SF2/ARv7 axis in the *in vivo* studies, using small compounds, like ASC-J9^®^, to degrade ARv7 may have a clinical advantage as ASC-J9^®^ has been proven to have low toxicity [26, 41] and high bio-stability [42] to suppress CRPC. Preclinical studies using various mouse models receiving ASC-J9^®^ treatment showed no obvious adverse effects with normal libido or fertility [41, 43, 44]. Furthermore, ASC-J9^®^ can be effectively delivered to PCa tissues in mice 6 hours after intro-peritoneal injection and the drug is stably retained in the PCa tissues to allow 48 hours interval injections [42].

The unexpected finding of another ARv7 degradation enhancer, Cisplatin, an FDA-approved drug that is currently used as chemotherapy for several tumors [45–48], has significant clinical implication. The positive results from preclinical studies in multiple mouse studies showing Cisplatin at low-doses can increase Enz sensitivity *via* degrading ARv7 will allow quick transitions into human clinical trials to further increase the Enz sensitivity to suppress CRPC cell growth in EnzR or DocR patients.

In conclusion, Doc-resistance is a common and negative outcome in clinical chemotherapy of CRPC. Our findings indicate Doc-chemotherapy in current clinical use prior to Enz treatment in CRPC patients might result in adverse effects of accelerating development of Enz resistance through enhanced ARv7 expression. We provide evidence that inhibition of the MALAT1/SF2/ARv7 axis in DocR CRPC cells can restore subsequent Enz sensitivity. Combining Enz treatment with shARv7, siMALAT1, shSF2, or using novel ARv7 degradation enhancers: Cisplatin or ASC-J9^®^, may help prevent adverse effects induced by Doc to extend CRPC patients’ survival.

## MATERIALS AND METHODS

### Generation of Doc-resistant (DocR) prostate cancer (DocRPC) cell models

The PCa cell lines CWR22Rv1 and C4-2 were obtained from American Type Culture Collection (ATCC, Manassas, VA, USA) and maintained in RPMI 1640 media (GIBCO, Waltham, MA, USA) supplemented with 10% FBS. DocR clones, DocR1_CWR22Rv1, and DocR3_C4-2 were selected by culturing cells with Doc. To develop Doc-resistance, the parental Doc-sensitive (DocS) PCa cells were treated starting with a dose of 0.1 nM Doc. The surviving cells were repeatedly collected and cultured with a higher Doc dosage (increased by 0.1 nM each time and up to 2 nM), with two to four weeks per dose. DocR1_CWR22Rv1 and DocR3_C4-2 cells developed Doc-resistance after 8 months for DocR1_CWR22Rv1 and 9 months for DocR3_C4-2 cells. The stable DocRPC cells were continuously treated with 2 nM Doc to maintain Doc-resistance. The DocR2_CWR22Rv1 cells were a gift from Dr. Carlos Cordon-Cardo in Mt. Sinai Hospital, the DocR2_CWR22Rv1 cells have been selected for 2 years and have different gene expression profiles from DocR1_CWR22Rv1.

### Cell proliferation assay

To test Enz-resistance development, DocR cells were seeded in 24-well plates (5x10^3^ cells/ 500 μl media per well) with Doc-containing media for 24 hr. All wells were gently washed by 1x PBS and then treated with Enz (day 0). Cells were then prepared for MTT assays on days 0, 2, 4, and 6. Absorbance values of a growth curve greater than the control indicate Doc-resistance, while equal or lower values suggest Doc susceptibility.

### Reporter gene assay

The promoter of *MALAT1*, (-2000 to ~+1) was cloned into the pGL3-basic luciferase vector and named as p*MALAT1*-luc. Then 200 ng pWPI-AR and 200 ng p*MALAT1*-luc plus 2 ng pRL-TK were transfected into 1x10^5^ C4-2 cells with lipofectamine 3000 for 24 hrs. After transfection, C4-2 cells were incubated with 1 nM DHT and treated with 0, 0.5, or 1 nM Doc for another 24 hrs, then lysed by commercial lysis buffer and analyzed with Promega dual-luciferase reporter assay system. Renilla-luciferase (protein product from pRL-TK) was used as transfection efficiency control.

### Chemical compounds

ASC-J9^®^ (IUPAC Name: (1E,4Z,6E)-1,7-bis(3,4-dimethoxyphenyl)-5-hydroxyhepta-1,4,6-trien-3-one) was purchased from AndroScience Corp. (San Diego, CA). Enzalutamide (IUPAC Name: 4-(3-(4-cyano-3-(trifluoromethyl)phenyl)-5,5-dimethyl-4-oxo-2-thioxoi-midazolidin-1-yl)-2-fluoro-N-methylbenzamide) was purchased from MedChem Express (Monmouth Junction, NJ, USA). Cisplatin (IUPAC Name (SP-4-2)-diamminedichloroplatinum (II)) was purchased from VWR (Radnor, PA, USA). Docetaxel (IUPAC Name: 1,7β,10β-trihydroxy-9-oxo-5β,20-epoxytax-11-ene-2α,4,13α-triyl 4-acetate 2-benzoate 13-{(2R,3S)-3-[(tert-butoxycarbonyl)amino]-2-hydroxy-3-phenylpropanoate}) was purchased from LC Laboratories (Woburn, MA, USA).

### Gene interruption by lenti-shRNA or siRNA

PLKO.1-puro-shARv7 and PLKO.1-puro-shSF2 were constructed with target sequence 5′-GCGAGTAACAAGGGCATGGAA-3′ and 5′-CTTCAAGGTTTCGAGAGTTAA-3′, respectively, according to Addgene's pLKO.1 protocol. Lentiviral particles were generated by calcium phosphate transfection of lentivirus expressing plasmids, packaging plasmid psPAX2, and envelope plasmid pMD2.G into HEK 293T cells and lentiviral particles were collected to infect target cells according to Add gene's pLKO.1 protocol. The siMALAT1 was purchased from IDT. The transfection procedure was according to the instructions.

### RNA extraction and quantitative Real-Time PCR analysis

Total RNAs were isolated using Trizol reagent (Invitrogen, Grand Island, NY) and 2 μgs of total RNA was subjected to reverse transcription using Superscript III transcriptase (Thermo Fisher Scientific, Waltham, MA USA). Quantitative real-time PCR (qRT-PCR) was conducted using a Bio-Rad CFX96 system with SYBR green to determine mRNA expression levels of genes of interest. Expression levels were normalized to expression of *GAPDH*. (All primer sequences were listed in Supplementary Figure 6).

### Western blotting

Cells were lysed in lysis buffer and proteins (50 μg) were separated on 10% SDS/PAGE gel and then transferred onto PVDF membranes (Millipore, Billerica, MA). After blocking membranes with 5% non-fat milk, they were incubated with appropriate dilutions of specific primary antibodies anti-AR (N-20, #sc-816; SCBT, Dallas, TX, USA), anti-ARv7 (#ab198394, Abcam, Cambridge, UK), anti-SF2 (#ab38017, Abcam), anti-GAPDH (#sc-48166, SCBT), or anti-α-tubulin (#sc-8035, SCBT). The blots were then incubated with HRP-conjugated secondary antibodies (goat anti-rabbit #G21234 and goat anti-mouse #G21040, Invitrogen) and visualized using the ECL system (Thermo Fisher Scientific).

### Generation of Enz-resistant (EnzR) DocRPC cell models

For EnzR clones, the DocR1_CWR22Rv1, DocR2_CWR22Rv1 and DocR3_C4-2 cells were selected by culturing cells with 20 μM Enz for 1-2 months and then prepared for MTT assays to analyze cells proliferation rates to check for resistance development.

### Prostate orthotopic implantation

Male 6-8-week-old nude mice were purchased from NCI (Bethesda, MD, USA). The Matrigel mixture with 1-2x10^6^ luciferase labeled PCa (DocS1, DocR1, DocR1+shControl, or DocR1+shARv7) cells were orthotopically injected into both anterior prostates. After 2 weeks of implantation, each set of mice were randomly assigned into experimental groups and treated with DMSO, 35 mg/kg Enz, 75 mg/kg ASC-J9^®^, or the combination of Enz+ASC-J9^®^, by i.p. injection 3x/wk/2wks. Tumors were monitored weekly by In Vivo Imaging System (IVIS).

### Statistics

Experiments were performed at least 3 times with data points in triplicate. Statistical analyses were carried out with Prism 6. The data values were presented as mean ± SD. Differences in mean values between two groups were analyzed by two-tailed Student’s *t* test or ANOVA. *p* ≤ 0.05 was considered statistically significant.

## Supplementary Material

Supplementary Figures
