# Supplementary material for: Preclinical studies show using enzalutamide is less effective in docetaxel-pretreated than in docetaxel-naïve prostate cancer cells
Source: Aging (Albany NY). 2020 Sep 10;12(17):17694–712. doi: 10.18632/aging.103917 (PMC7521536; doi:10.18632/aging.103917)
Supplement: Supplementary Figures [file aging-12-103917-s001..pdf]

SUPPLEMENTARY FIGURES

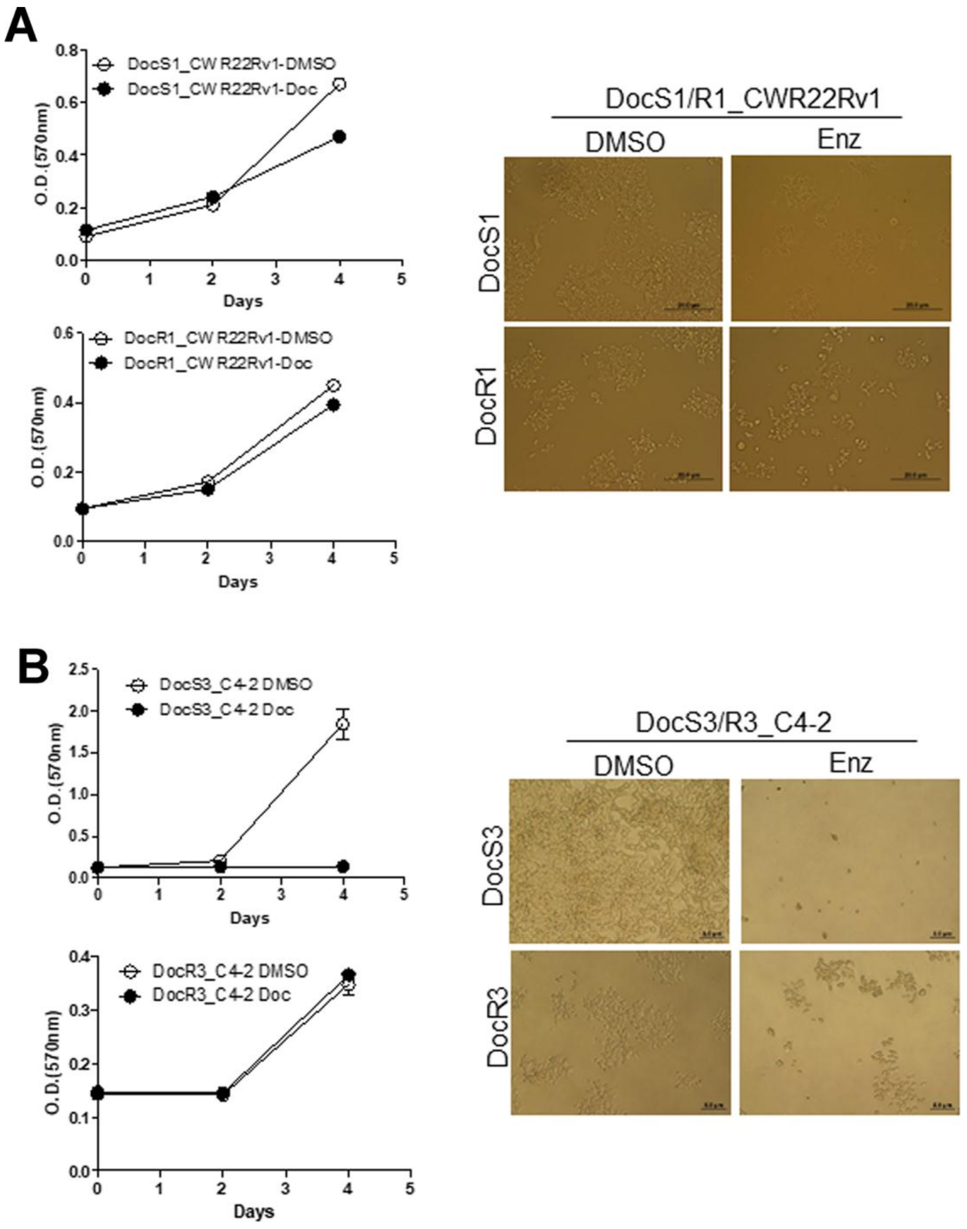

Supplementary Figure 1. Acquired docetaxel resistance in CRPC cells could facilitate the development of cross-resistance to Enz treatment. (**A**) DocR1_CWR22Rv1 and (**B**) DocR3_C4-2 (lower panels) cells are more resistant to Doc treatment than DocS1_CWR22Rv1 and DocS3_C4-2 cells (upper panels). The cell viability (left) and morphological changes (right) after treating with 20 µM of Enz for one week.

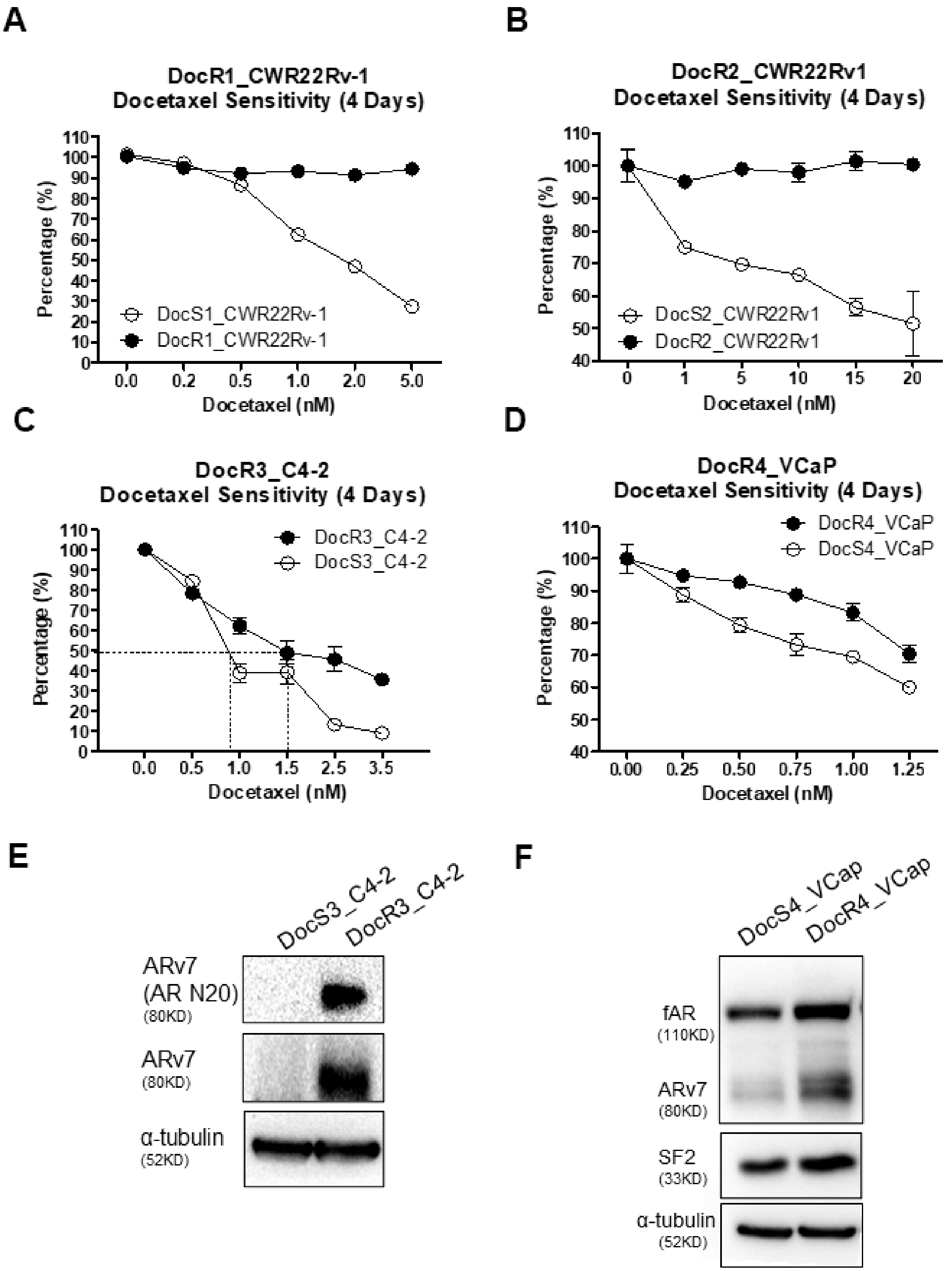

Supplementary Figure 2. ARv7 is increased in DocR CRPC cells. (**A**–**D**) The Docetaxel sensitivity of (**A**) DocS1_CWR22Rv1 cells vs. DocR1_CWR22Rv1 cells, (**B**) DocS2_CWR22Rv1 cells vs. DocR2_CWR22Rv1 cells (**C**) DocS3_C4-2 cells vs. DocR3_C4-2 cells and (**D**) DocS4_VCaP vs. DocR4-VCaP. (**E**, **F**) The DocR cells express higher ARv7. ARv7 protein level (**E**) in DocS3_C4-2 vs. DocR3_C4-2 and (**F**) in DocS4_VCap or DocR4_VCap cells.

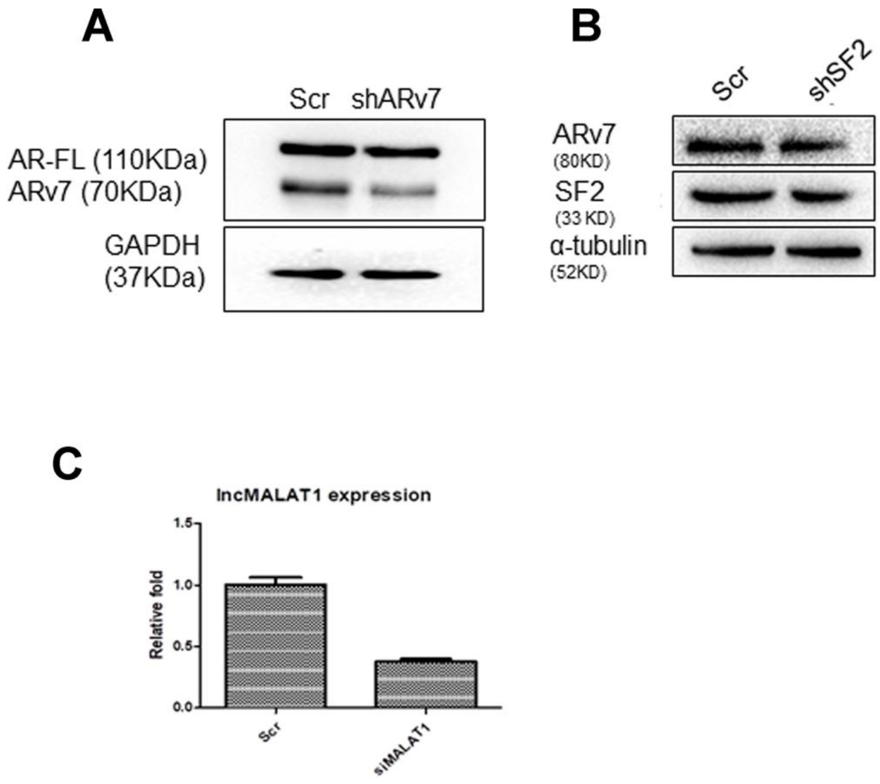

Supplementary Figure 3. The Knock down efficiency of (**A**) ARv7, (**B**) SF2, and (**C**) MALAT1.

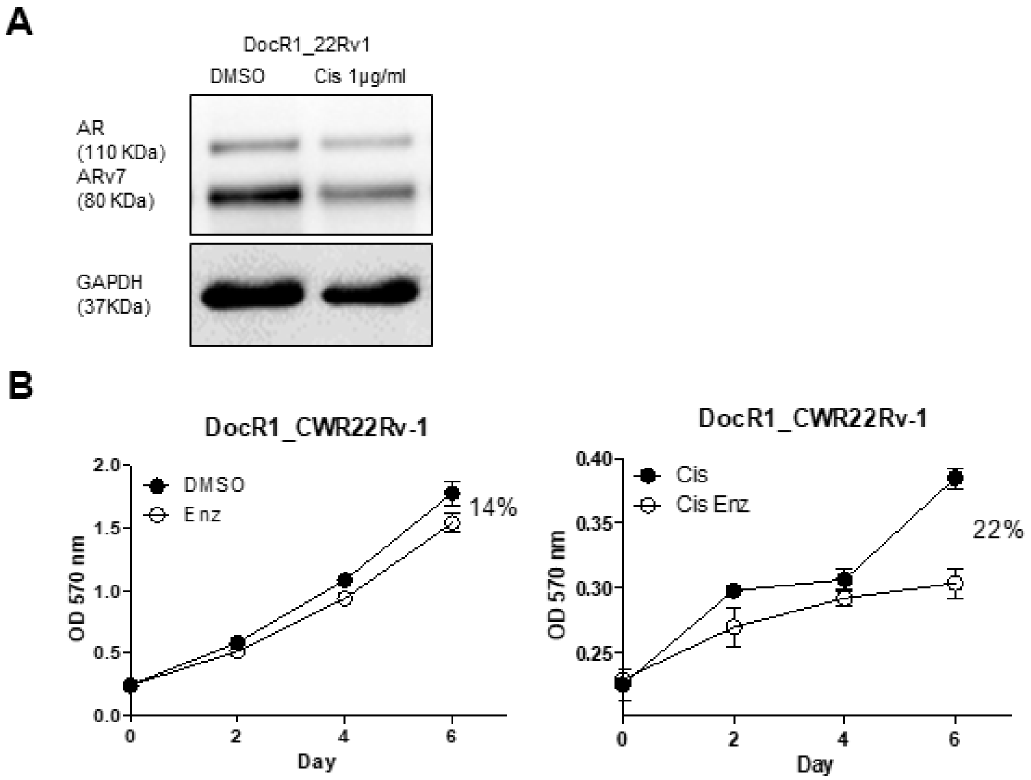

Supplementary Figure 4. The (**A**) protein level of ARv7 and (**B**) Enz Sensitivity in DocR1_CWR22Rv-1 cells after cisplatin treatment.

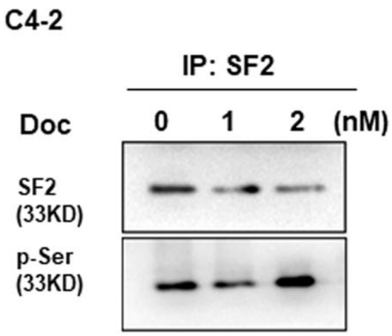

Supplementary Figure 5. Transient Doc treatment increases the phosphorylation of SF2 (p-Ser) in C4-2 cells. C4-2 cells were treated with 0, 1, or 2 nM Doc for 24 hrs. Protein extracts were examined with anti-SF2 (upper) and phosphorylated SF2 (p-Ser SF2; bottom) using immunoblot.

GAPDH Forward	5'-TGTGGGCATCAATGGATTTGG-3'
GAPDH Reverse	5'-ACACCATGTATTCCGGGTCAAT-3'
ARv7 Forward	5'-CCATCTTGTCGTCTTCGGAAATGT-3'
ARv7 Reverse	5'-TTTGAATGAGGCAAGTCAGCCTTTCT-3'
SRSF2 Forward	5'-CCCGATGTGGAGGGTATGAC-3'
SRSF2 Reverse	5'-GAGACTTCGAGCGGCTGTAG-3'
MALAT1 Forward	5'-CGGAAGTAATTCAAGATCAAGAG-3'
MALAT1 Reverse	5'-ACTGAATCCACTTCTGTGTAGC-3'

Supplementary Figure 6. The primer sequences for quantitative PCR analyses.
